# Re: Evaluating RAG-LLMs for Infective Endocarditis Prophylaxis: Accuracy and Efficiency

**DOI:** 10.1016/j.identj.2026.109528

**Published:** 2026-03-25

**Authors:** Carlos Fernando Mourão, Luiz Eduardo Juliasse

**Affiliations:** Department of Basic and Clinical Translational Sciences, Tufts University School of Dental Medicine, Boston, Massachusetts, USA

Dear Editor,

Rewthamrongsris et al. report a counterintuitive finding that warrants closer attention: retrieval-augmented generation (RAG) reduced accuracy in Claude 3.5 Sonnet and Gemini 1.5 Pro relative to their native counterparts, while yielding no statistically significant improvement across models overall.^1^ This pattern—where a technique widely regarded as beneficial degraded performance in high-performing models—underscores 3 interpretive and translational challenges that we believe merit direct discussion.

First, the marked variability across models and preprompting strategies reflects a fundamental validation problem: large language model (LLM) performance is sensitive to the entire *“prompted system,”* which includes prompt text, retrieval configuration, and generation parameters. Reporting standards for LLM-based research are now available and explicitly require transparent specification of intended use, system instructions, retrieval settings, and inference parameters.^2^^,^^3^ Because the same preprompt text produces qualitatively different effects across architectures, publishing only the prompt string—without the surrounding system configuration—limits reproducibility and cross-study comparability. We encourage the authors and future investigators to report complete system specifications alongside robustness checks, including repeated runs and prompt-sensitivity analyses,^3^^,^^4^ to help readers distinguish architecture-specific behavior from prompt-specific artifacts.

Second, the pilot’s educational findings would gain considerably greater translational value from a *guideline-only* control arm—that is, participants given direct access to the 2021 American Heart Association document without any LLM intermediary. Without this comparator, it is impossible to determine whether the observed changes in accuracy and time penalties stem from the LLM’s synthesis capabilities, the cognitive load of interfacing with a chatbot, or simply the act of consulting any external resource. This design gap is particularly consequential, given that DeepSeek Reasoner’s average response latency of approximately 40 seconds per query imposed a cumulative waiting period of nearly seven minutes per participant—a cost that guideline-only access would not incur. Establishing this benchmark in future work would clarify whether LLM assistance offers genuine added value over conventional clinical reference retrieval.

Third, overall accuracy is an incomplete safety signal for high-stakes clinical recommendations. A model achieving 83% accuracy may still produce clinically critical errors with highly asymmetric consequences. For infective endocarditis prophylaxis, misclassifying a patient with a prosthetic cardiac valve or a history of endocarditis as low-risk—and therefore withholding antibiotic prophylaxis—carries a different magnitude of harm than erroneously recommending prophylaxis for a low-risk patient. Future evaluations would benefit from a clinically oriented error taxonomy that distinguishes under treatment from overtreatment errors, stratified by cardiac risk group and specific antibiotic regimen questions, and from outcome measures that assess decision impact rather than item-level correctness alone.^5^^,^^6^

This work makes a valuable contribution by showing that RAG is neither uniformly beneficial nor deployable without careful model selection. Translating these findings into safe chairside and educational practice will require complete prompted-system reporting, robustness testing,^2^, ^3^, ^4^ guideline-access benchmarks to isolate the incremental value of LLM synthesis, and clinically meaningful error analyses that go beyond aggregate accuracy.^5^^,^^6^ Above all, the authors’ conclusion bears emphasis: given that infective endocarditis is a life-threatening condition, LLM outputs must always be independently verified before clinical application.^1^

## Declaration of generative AI and AI-assisted technologies in the manuscript preparation process

During the preparation of this work, the author(s) used Grammarly to polish the grammar of this letter. After using this tool/service, the author(s) reviewed and edited the content as needed and take(s) full responsibility for the content of the published article.
